# Novel Genetic Rearrangements Termed “Structural Variation Polymorphisms“ Contribute to the Genetic Diversity of Orthohepadnaviruses

**DOI:** 10.3390/v11090871

**Published:** 2019-09-17

**Authors:** Kei Fujiwara, Kentaro Matsuura, Kayoko Matsunami, Etsuko Iio, Yoshihito Nagura, Shunsuke Nojiri, Hiromi Kataoka

**Affiliations:** Department of Gastroenterology and Metabolism, Nagoya City University Graduate School of Medical Sciences, Nagoya, Aichi 467-8601, Japan

**Keywords:** orthohepadnavirus, hepatitis B virus, genetic diversity, structural variation

## Abstract

The genetic diversity of orthohepadnaviruses is not yet fully understood. This study was conducted to investigate the role of structural variations (SVs) in their diversity. Genetic sequences of orthohepadnaviruses were retrieved from databases. The positions of sequence gaps were investigated, since they were found to be related to SVs, and they were further used to search for SVs. Then, a combination of pair-wise and multiple alignment analyses was performed to analyze the genomic structure. Unique patterns of SVs were observed; genetic sequences at certain genomic positions could be separated into multiple patterns, such as no SV, SV pattern 1, SV pattern 2, and SV pattern 3, which were observed as polymorphic changes. We provisionally referred to these genetic changes as SV polymorphisms. Our data showed that higher frequency of sequence gaps and lower genetic identity were observed in the pre-S1-S2 region of various types of HBVs. Detailed examination of the genetic structure in the pre-S region by a combination of pair-wise and multiple alignment analyses showed that the genetic diversity of orthohepadnaviruses in the pre-S1 region could have been also induced by SV polymorphisms. Our data showed that novel genetic rearrangements provisionally termed SV polymorphisms were observed in various orthohepadnaviruses.

## 1. Introduction

Hepatitis B virus (HBV) infection is an important global health issue and can cause acute and chronic liver diseases. An estimated 257 million people are deemed to be infected with HBV as they are positive for hepatitis B surface antigen (HBs-Ag), and HBV infection causes approximately 900,000 deaths per year from complications such as liver cirrhosis and hepatocellular carcinoma [1]. In addition, fatal liver failure caused by reactivation of occult HBV in HBs-Ag-negative patients, who were considered as cured of a previous infection, by treatment with anti-cancer and immune-modulating drugs is a serious clinical problem [2,3]. Therefore, HBV infection is a lifelong threat even after clinical cure of chronic infection.

HBV is a member of the hepadnaviridae family, which is composed of two genera—the genus Orthohepadnavirus infects mammals, and the genus Avihepadnavirus infects birds [4]. Previously, HBVs that infect non-human primates such as gorilla, chimpanzee, gibbon, orangutan, and woolly monkey (WMHBV) [5,6,7,8,9,10], and rodents, such as woodchuck (WHV), ground squirrel (GSHV), and arctic squirrel (ASHV), have been reported [11]. Recently, bat HBVs (tent-making bat HBV (TBHBV), Pomona bat HBV (PBHBV), unspecified bat HBV from China (BHBV-C), long-fingered bat HBV (LBHBV), horseshoe bat HBV (HBHBV), roundleaf bat HBV (RBHBV)) have been reported as new members of orthohepadnaviruses [12,13,14]. Since bat HBVs are recent discoveries, reports on the detailed genetic analysis of these species of orthohepadnaviruses are limited.

Previous reports have suggested that the complexity of genetic diversity of HBVs is related to their mosaic genome structure [15,16,17], and Littlejohn et al. [4] proposed that complex genetic changes are also important for HBV genetic diversity in addition to an accumulation of nucleotide substitutions. Presently, the genetic diversity of orthohepadnaviruses is not fully understood. Recent advances in genome research have elucidated non-canonical forms of genetic rearrangements called complex structural variations (SVs) [18,19,20]. Quinlan and Hall [19] defined complex SVs as variants with multiple breakpoints whose origin cannot be explained by a single end-joining event. Yalcin et al. [20] reported complex SVs as two or more SVs co-occurring at the same locus. With the discovery of a very rare and unusual HBV strain, complex SVs in HBV were described [21,22,23]. The concept of complex SVs was entirely novel in HBV genome research, and hidden genetic changes were elucidated by the analysis of the complex SVs. Therefore, based on the above-mentioned studies that previously reported complex SVs in human HBVs, we extended our study to analyze the genetic diversity of different species of orthohepadnaviruses by focusing on SVs. In our present research, novel unique forms of SVs were frequently observed. We found that genetic sequences at certain positions of the genome of different species of orthohepadnaviruses maintained relatively high sequence identities, which were observed on either side of low identity sequences showing multiple patterns including no SV, SV pattern 1, SV pattern 2, and SV pattern 3. These changes were observed as polymorphic differences. We provisionally described these genetic changes as SV polymorphisms. We report here evidence demonstrating that SV polymorphisms contributed to the genetic diversity of orthohepadnaviruses.

## 2. Materials and Methods

### 2.1. Reference Sequences 

Consensus reference sequences and original datasets of human and non-human primate HBVs are described in Fujiwara et al. [22]. Information on the genetic sequences of human and non-human primate, bat (TBHBV, PBHBV, BHBV-C, LBHBV, HBHBV, and RBHBV), and rodent (WHV, GSHV, and ASHV) HBVs were retrieved from PubMed or searched directly from Genbank/EMBL/DDBJ and compiled in Table S1.

### 2.2. Phylogenetic Analysis

Phylogenetic analysis was performed with the MEGA software version 6 [24], using neighbor-joining method. Bootstrap resampling and reconstruction with 1000 replicates were carried out. Genetic distance calculation and pair-wise distance comparisons were performed using Kimura two-parameter model integrated into the MEGA software.

### 2.3. Sequence Gap Analysis

Human HBV genotypes A to H, non-human primate HBVs (orangutan, chimpanzee, gorilla, and gibbon), bat HBVs, and rodent HBVs listed in Table S1 were aligned with the reference sequence of WMHBV (AF046996) using MAFFT [25]. Sequence gap positions and gap lengths (insertion, +; deletion, -) were manually determined.

### 2.4. Sequence Identity Analysis

The sequence identity (%) among the complete genomes and each ORF of WMHBV, bat HBVs, and rodent HBVs were determined by MAFFT [25]. Three strains from each species were used when available. The strains used in this analysis are underlined in Table S1. To investigate partial sequence identity in the pre-S1 to S2 region of WMHBV, bat HBVs, and rodent HBVs, pair-wise alignments of the WMHBV, six bat HBVs, and three rodent HBVs were performed using MAFFT [25]. Forty-five patterns of pair-wise data were obtained as shown in Figure 3 and Figure S2A,B. Maintenance of sequence identity was arbitrarily defined as equal to or more than 65% sequence identity and nucleotide length of equal to or more than 10 nts without sequence gaps.

### 2.5. Multiple Alignment Analysis

Multiple alignment comparisons were performed using MAFFT ([25]. The repeated multiple alignment analysis of WMHBV and bat HBV sequences in the pre-S region (Figure 4A) was conducted based on the data obtained by pair-wise analysis of the 45 patterns shown in Figure 3 and Figure S2A,B.

### 2.6. Sequence Similarity Search

Similarity searches were performed using NCBI BLAST 2.2.31 [26]. 

## 3. Results

### 3.1. The Genetic Sequences of Orthohepadnaviruses

Together with WMHBV and rodent HBVs (WHV, GSHV, and ASHV), bat HBVs (TBHBV, PBHBV, BHBV-C, LBHBV, HBHBV, and RBHBV) and species of primate HBVs were analyzed in this study (Table S1). Phylogenetic analysis using the complete genomes of all the investigated orthohepadnaviruses showed similar patterns as demonstrated in a previous report [12] (Figure S1), suggesting the strains of orthohepadnaviruses used in this study were phylogenetically similar to those analyzed in the previous study.

### 3.2. Analyses of the Sequence Gaps and Percentage Identities among WMHBV, Bat HBVs, and Rodent HBVs

**Figure 1:**
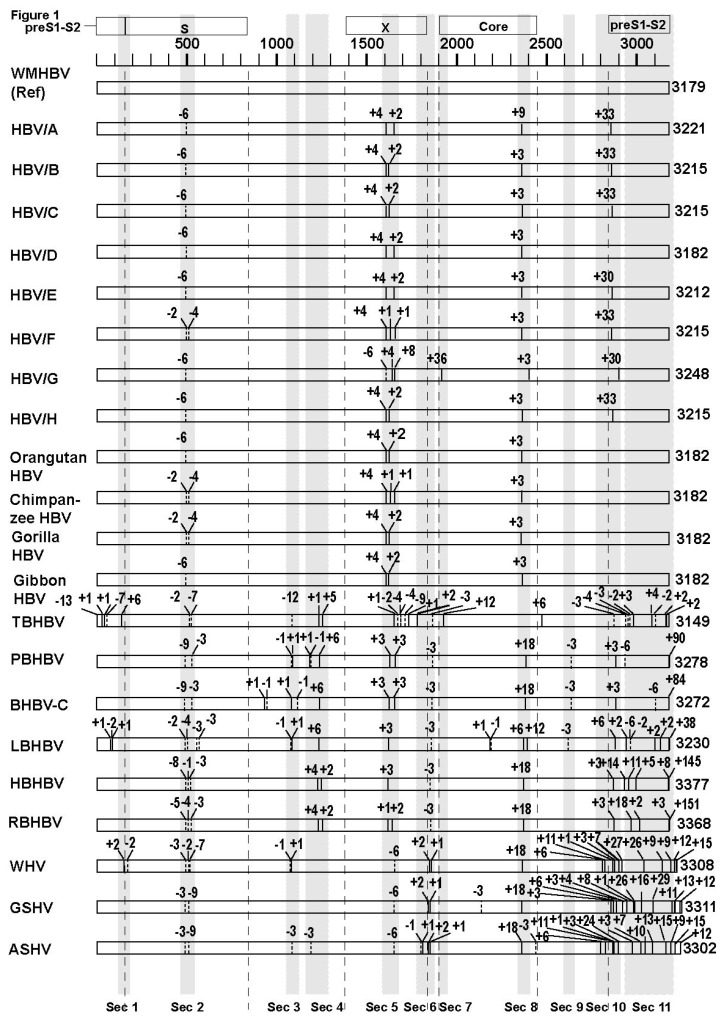
Comparison of sequence gaps. Each full genome sequence of human HBV genotypes A to H, primate HBVs (orangutan, chimpanzee, gorilla, and gibbon), bat HBVs (TBHBV, PBHBV, BHBV-C, LBHBV, HBHBV, and RBHBV), and rodent HBVs (WHV, GSHV, and ASHV) was aligned with the reference sequence of WMHBV (AF046996). Sequence gap positions and gap lengths (insertion, +; deletion, -) are shown. Areas with frequent sequence gaps among species are shown in shaded rectangles and named Sec 1 to Sec 11. Sec, section.

In previous studies, we found that complex SVs tended to accompany sequence gaps [21,22,23], thus, the positions and lengths of sequence gaps in bat and rodent HBVs were analyzed using WMHBV as a reference sequence in order to investigate the presence of SVs in their genomes. Pair-wise comparisons of the sequences of each species were performed by MAFFT ([25]. (Figure 1). The number of sequence gaps in the small S, X, Core, and pre-S1-S2 regions in the genetic sequences of bat and rodent HBVs was 23, 20, 14, 74 respectively, and sequence gaps were demonstrated to be more abundant in the pre-S1-S2 region than in the other regions (Table 1). Areas with frequent sequence gaps among species were indicated as section (Sec) 1 to 11 in Figure 1. Further, sequence identities in the full genome and the small S, X, Core, and pre-S1-S2 regions among WMHBV, bat HBVs, and rodent HBVs were analyzed. We found that the sequence identity in the pre-S1-S2 region was lower than that in the other regions and in the full genome (Table 1). Therefore, it was speculated that the higher frequency of sequence gaps and lower sequence similarities in the pre-S1-S2 region reflected the presence of SVs in the genomes of these orthohepadnaviruses.

### 3.3. Unique SVs in the Core and Pre-S1 Promoter to pre-S1 ORF Start Site Regions

**Figure 2:**
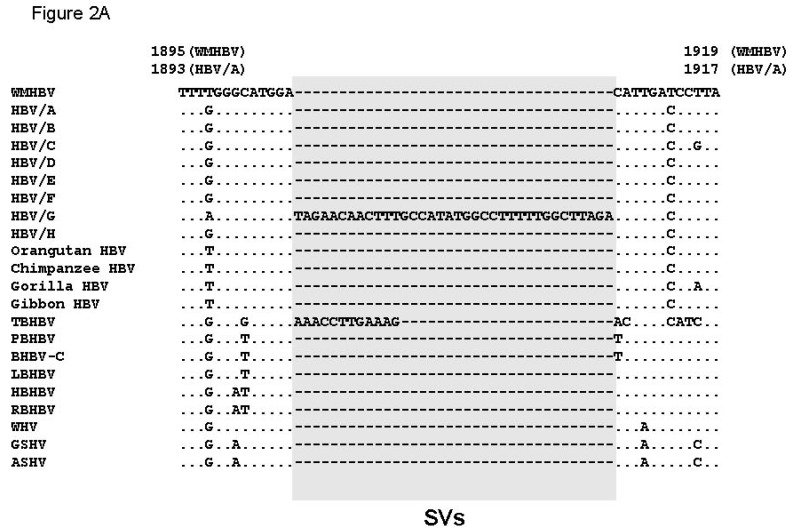

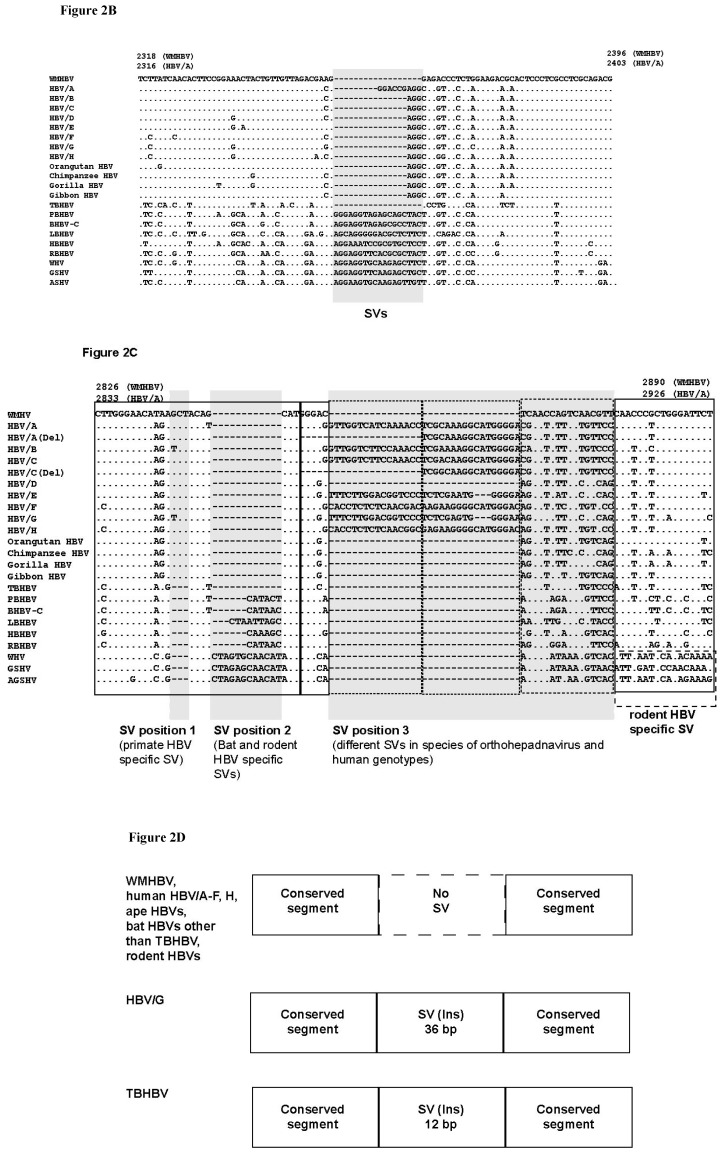

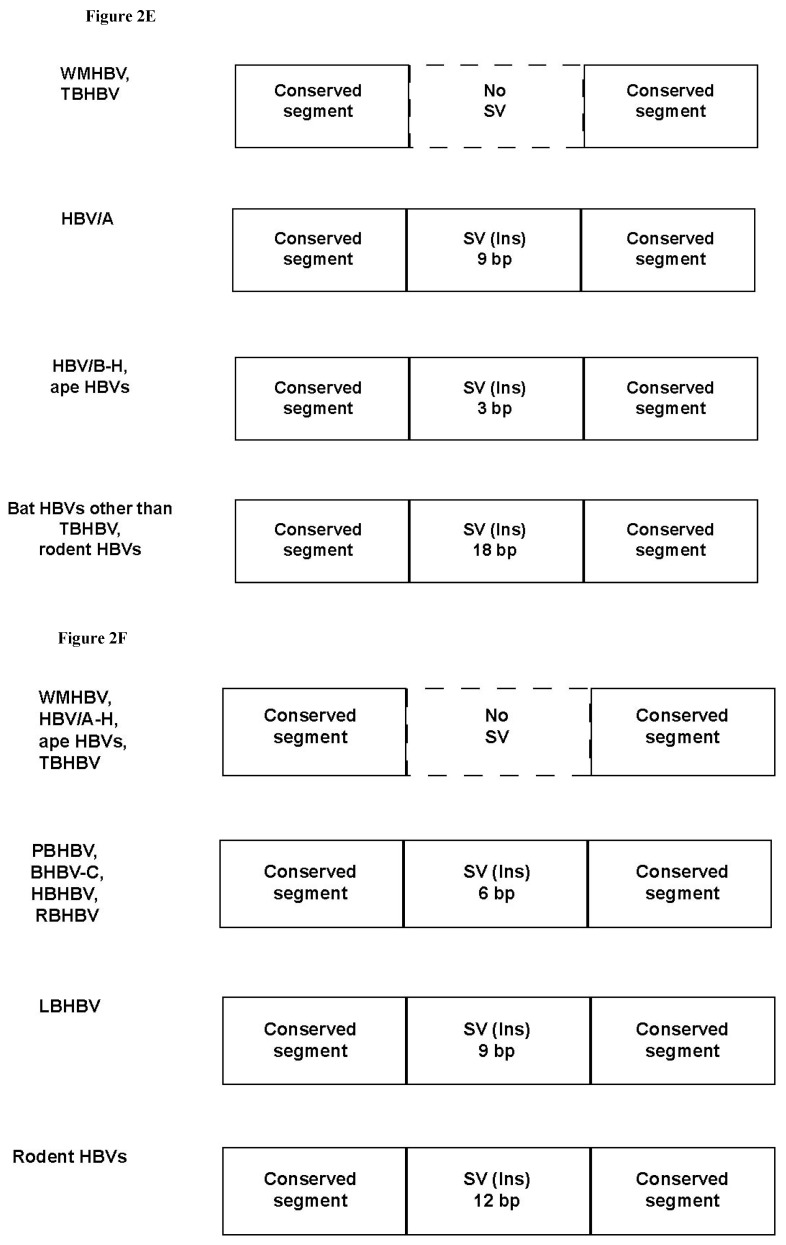

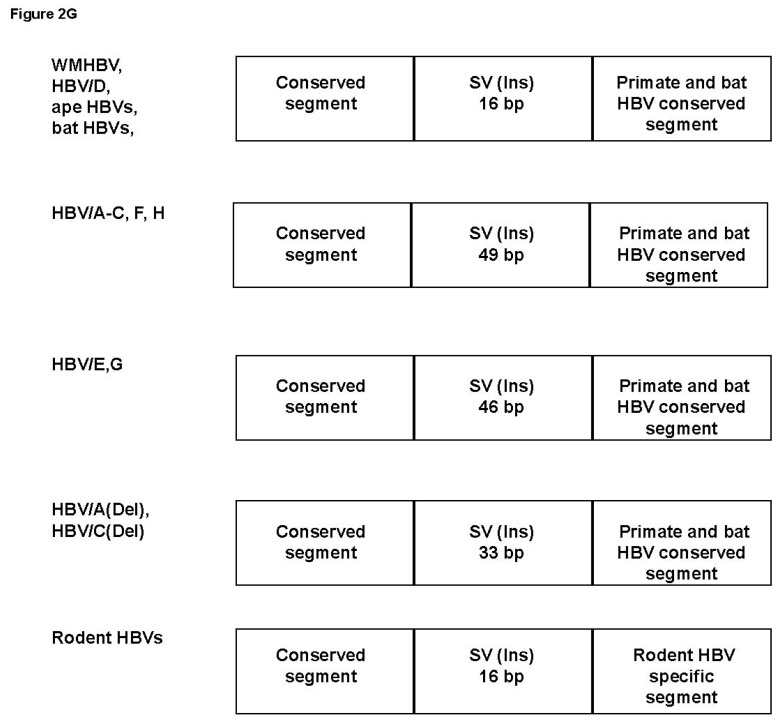
Analysis of structural variations (SVs) among orthohepadnaviruses. Ins, insertion. (**A**), Alignment of the Core ORF start region from nt 1895 to 1919 (WMHBV) revealed different SVs in HBV/G and TBHBV as shown in shaded rectangle. (**B**), Alignment of the Core region from nt 2318 to 2396 (WMHBV) revealed different SVs in several orthohepadnaviruses as shown in shaded rectangles. (**C**), Alignment of the pre-S1 ORF start site region from nt 2826 to 2890 (WMHBV) revealed different SVs in species with orthohepadnavirus. Since this area contains multiple segments with SVs, locations of SVs are shown as SV position 1 to 3 as shown in shaded rectangles. (**D**), The SV polymorphisms observed in Figure 2A is illustrated in a simplified manner. (**E**), The SV polymorphisms observed in Figure 2B is illustrated in a simplified manner. (**F**), The SV polymorphisms observed in SV position 2 of Figure 2C is illustrated. (**G**), The SV polymorphisms observed in SV position 3 of Figure 2C is illustrated.

In order to identify genetic variations that could have been caused by SVs in the genetic sequences of these orthohepadnaviruses, the areas with frequent sequence gaps as shown in section (Sec) 1 to 11 in Figure 1 were investigated. Genetic sequence alignment search of orthohepadnaviruses revealed SVs in Sec 7, 8, and 10. Sec 11 showed highly complicated alignments, and SVs were not identified in the initial search. Sec 7 showed unique SVs in HBV genotype G (HBV/G) and TBHBV. It was previously reported that the human HBV/G has a 36-bp insertion of unknown origin in its Core ORF [27], and in this study, it was revealed that TBHBV has a 12-bp insertion at the same position (Figure 2A). The insertions of HBV/G and TBHBV, existing as polymorphic genetic variations, showed low sequence identity with each other, suggesting that they were unrelated. In order to obtain more information on these inserted sequences, BLAST [26] searches were performed, but the origins of these sequences could not be further clarified.

Sec 8 also showed unique variation among the different species of orthohepadnaviruses. A previous study has shown that HBV/A has a 6-bp insertion in its Core ORF [28]. We show here that this position is a hotspot of polymorphic genetic variability. Although WMHBV and TBHBV do not have any insertions, in addition to the 9-bp insertion in HBV/A, other human HBVs have 3-bp insertions, and bat and rodent HBVs have 18-bp insertions at this position (Figure 2B). 

We next analyzed Sec 10, which is located around the pre-S1 promoter to pre-S1 ORF start site in our repertoire of orthohepadnaviruses. Different SVs in human and non-human primate HBVs have been previously reported in this area [22]. Our data showed that SVs specific to primate, bat, rodent, and human HBVs were observed (Figure 2C). In particular, LBHBV, non-LBHBV bat HBVs, and rodent HBVs contain different SVs with low sequence identities and sequence gaps (SV position 2 in Figure 2C). These data suggest that they have a similar form of SVs as those observed in the Core region. In addition, human HBVs have various SVs in this area, whereas other orthohepadnaviruses have 16-bp insertions as shown in SV position 3 of Figure 2C, which imply that the different genotypes of human HBVs have high genetic variability due to the presence of different SVs. All the analyzed SVs in the Core and pre-S1 promoter to pre-S1 ORF start site regions contain similar patterns of SVs. Genetic sequence identities were relatively conserved at certain positions of the genome, which were found to bound on either side low identity sequences that could be separated into multiple SVs: no SV, SV pattern 1, SV pattern 2, and SV pattern 3. We provisionally defined these genetic changes as SV polymorphisms, and they were frequently observed as important polymorphisms among the different species of orthohepadnaviruses. All of the SV polymorphisms that we found as shown in Figure 2A–C are illustrated in a simplified manner in Figure 2D–G.

### 3.4. Analysis of the Pre-S1-S2 Region

**Figure 3:**
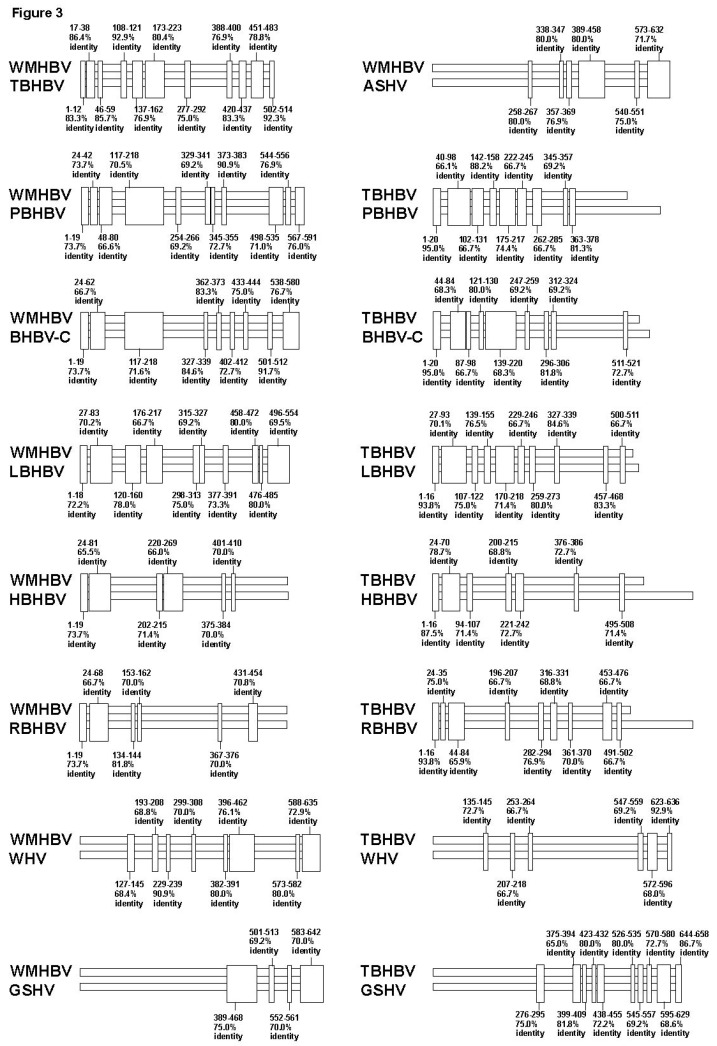
Pair-wise alignments and comparisons of WMHBV, six bat HBVs, and three rodent HBVs in pre-S1 to S2 regions produced 45 patterns, of which the first 16 are shown. (The remaining 29 patterns are shown in Figure S2A,B.) This analysis was performed to clarify partial sequence identity between two species, which may indicate covert conserved genetic segments. Consensus sequence of each species was used for analysis. Maintenance of sequence identity was defined as having approximately 65% identity without gaps and a genetic sequence length ≥ 10 bps without sequence gaps, and segments satisfying these conditions are shown in vertical rectangles. Numbers above or below rectangles show the nucleotide positions where high % identity was observed in pair-wise alignment, and % identity of segments.

**Figure 4:**
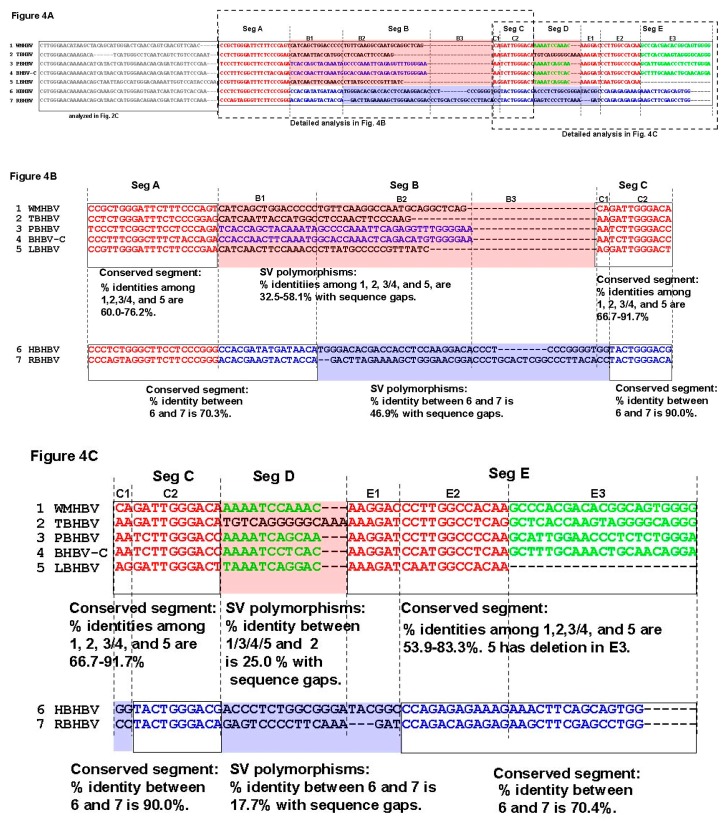
SV polymorphisms in part of pre-S1 region. WMHBV, TBHBV, PBHBV, BHBV-C, LBHBV, HBHBV, and RBHBV are shown as 1, 2, 3, 4, 5, 6, and 7, respectively, in the data analysis. (**A**), Alignment of part of the pre-S1 region. The multiple alignment data were analyzed using the data of sequence identities shown in Figure 3 and Figure S2A,B. The sequences were divided into five segments (Seg A to E) and additional sub-segments. PBHBV and BHBV-C showed high % identity in this region. The partial sequence segments with high identities obtained by pair-wise analysis (shown in Figure 3 and Figure S2A,B) are colored. Segments with relatively high % identities maintained among WMHBV and bat HBVs or WMHBV/TBHBV/PBHBV/BHBV-C/LBHBV are shown in red colored letters. Segments with relatively high % identities maintained between PBHBV and BHBV-C or HBHBV and RBHBV are shown in blue colored letters. Segment with relatively high % identities maintained among WMHBV and some of bat HBVs are shown in lime colored letters. SV polymorphisms observed in WMHBV, TBHBV, PBHBV, BHBV-C, and LBHBV are surrounded by red shaded rectangles. SV polymorphisms observed in HBHBV and RBHBV are surrounded by blue shaded rectangles. The consensus sequence of each species was used for analysis. Seg, segment. (**B**), The detailed analyses of SV polymorphisms observed in Seg B in WMHBV, TBHBV, PBHBV/BHBV-C, LBHBV, and those observed in Seg B2+B3+C1 in HBHBV and RBHBV are shown. Conserved segments are shown in black-framed rectangles. Color letters and colored rectangles show same sequences and SVs as explained in Figure 4A. (**C**), The detailed analyses of SV polymorphisms observed in Seg D among WMHBV/PBHBV/BHBV-C/LBHBV and TBHBV, and those observed in Seg D+E1 between HBHBV and RBHBV are shown. Colored letters and rectangles show same sequences and SVs as explained in Figure 4A.

Thus far, very unique SV polymorphisms were observed in the Core and pre-S1 promoter to pre-S1 ORF start site regions, as shown in Figure 2A–G. However, as demonstrated above, the area with the highest genetic variability in the orthohepadnavirus genomes corresponds to the preS1–S2 region (Figure 1, Table 1). Therefore, we speculated that additional SV polymorphisms, may exist in the pre-S1-S2 region, and are the cause of higher frequency of sequence gaps and lower sequence identity. However, the high complexity of the genetic sequences in Sec 11 (Figure 1) hampered us from identifying SVs with conventional multiple alignments analyses that were used in Sec 7, 8 and 10 in Figure 1. In these sections, conserved genetic segments were observed, and SVs were identified from those conserved genetic segments as shown in Figure 2A–C. On the other hand, conserved genetic segments were not found in Sec 11, therefore, the detection of SVs was not possible. We then hypothesized that relatively conserved genetic segments among species with orthohepadnavirus were mixed with specific SVs to a species or a group and form the complexity. We speculated that relatively conserved segments could be identified by repeating pair-wise analysis. To elucidate covert conserved genetic segments and SVs, we performed pair-wise alignments of the approximately 500–690 bp sequences from pre-S1 promoter (nt 2826 of WMHBV) to the pre-S2 region using MAFFT, and 45 patterns of alignment comparisons were obtained. Maintenance of sequence identity was provisionally defined as having approximately 65% identity without gaps (based on the results shown in Table 1) and a genetic sequence length ≥ 10 bps. The positions with sequence identity are shown in Figure 3 and Figure S2A,B. In the analysis, it was found that PBHBV and BHBV-C, HBHBV and RBHBV, and rodent HBVs had high % identities except for some parts (Figure S3A–C). For example, PBHBV and BHBV-C maintained more than 75.0% identity in most parts, although this decreased to less than 50.0% with sequence gaps between nt 305–369 (Figure S3A). HBHBV and RBHBV showed a mosaic pattern of high identity of more than 65.0% and low identity of less than 50.0%; in nt 78–164 and nt 330–414, segments of low identity with sequence gaps were observed (Figure S3B). Rodent HBVs also showed mosaic patterns, and in some parts, low identity with sequence gaps was observed as well (Figure S3C). The sequence identity found in Figure 3 and Figure S2A,B were further analyzed by multiple alignments of WMHBV and bat HBV sequences. Rodent HBVs were excluded because of low genetic identities to primate and bat HBVs. Each genetic sequence segment with sequence identity found in the pair-wise analyses was marked in the multiple alignments, and by repeated analyses, conserved areas, common SVs shared by several species, and unique SVs shared by two or three species were revealed as shown in colored letters in Figure 4A. Those structures were more complicated than the SVs observed in Figure 2A–C. The part of pre-S1 region shown in Figure 4A was divided into five segments (segment A to E) according to partial sequence identities found in Figure 3 and Figure S2A,B. In addition, sub-segments were used to identify SVs. Thus, our data suggest that extremely complicated arrangements caused by SV polymorphisms contributed to the genetic variability in this region of orthohepadnaviruses as shown in Figure 4A–C. The detailed analyses of SV polymorphisms observed in segment A to C among WMHBV, TBHBV, PBHBV, BHBV-C and LBHBV, and HBHBV and RBHBV are shown in Figure 4B. Data in Figure 4B showed that SV polymorphisms were observed in segment B among WMHBV, TBHBV, PBHBV/BHBV-C, and LBHBV. In addition, those were observed in segments B2+B3+C1 between HBHBV and RBHBV. WMHBV, TBHBV, PBHBV/BHBV-C, and LBHBV, and HBHBV and RBHBV had conserved segments in both sides of SV polymorphisms. WMHBV, TBHBV, PBHBV/BHBV-C, and LBHBV maintained 60.0–76.2% identities in segment A. All of sudden, sequence identities decreased to 32.5–58.1% with sequence gaps in segment B. In segment C, sequence identities returned to 66.7–91.7%. Similar changes in % identity were observed in HBHBV and RBHBV. The presence of SV polymorphisms caused the complexity in the multiple alignment. Furthermore, differences in length in conserved segments and SV poylmorphisms caused additional complexities. For example, the conserved segment was observed in segment A, and SV polymorphisms were observed in segment B1+B2 in WMHBV, TBHBV, PBHBV/ BHBV-C, and LBHBV. On the other hand, the conserved segments were observed in segments A+B1, and SV polymorphisms were observed in segment B2+B3+C1 in HBHBV and RBHBV. Furthermore, the detailed analyses of SV polymorphisms observed in segment C to E are shown in Figure 4C. Data in Figure 4C showed that SV polymorphisms were observed in segment D in WMHBV/PBHBV/BHBV-C/LBHBV, and TBHBV. In addition, those were observed in segment D+E1 in HBHBV and RBHBV. WMHBV, TBHBV, PBHBV/BHBV-C, and LBHBV maintained 66.7–91.7% identities in segment C and, suddenly, sequence identities decreased to 25.0% on average between WMHBV/PBHBV/BHBV-C/LBHBV and TBHBV in segment D. In segment E, % identities were recovered to 53.9-83.3%. Similar changes were observed in HBHBV and RBHBV in segment C to E. The presence of SV polymorphisms and differences in length of conserved and SV segments caused the additional complexity. Therefore, our results very clearly demonstrate that complicated mixtures of covert conserved segments and SV polymorphisms, and in addition to differences in their location between WMHBV/TBHBV/PBHBV/BHBV-C/LBHBV and HBHBV/RBHBV could be the cause of sequence gaps and low identities. It was speculated that more complicated genetic changes than the patterns shown in Figure 4A–C existed on the 3’ side of this region; however, the genetic sequence structures were not further clarified.

## 4. Discussion

Previous studies that analyzed human and non-human primate HBVs did not focus on sequence gaps since they are not frequent in these HBVs. Nucleotide substitutions and recombinations have been considered as main causes of genetic diversity of primate hepadnaviruses [29,30,31]. However, analyses of these genetic changes were not enough to elucidate the cause of genetic diversity of these viruses, and researchers have therefore described the genetic diversity and evolution of hepadnaviruses as elusive [4,32] or enigmatic [33]. In the present study, we focused on the importance of sequence gaps since the complex SVs in HBVs that we had discovered in previous studies [21,22,23] frequently contained sequence gaps. We first searched for sequence gaps in order to determine the association of SVs with genetic diversity of orthohepadnaviruses. We found many sequence gaps in bat and rodent HBVs and discovered that sequence gaps were accumulated in the preS1-S2 region as shown in Figure 1 and Table 1. Further, we found that sequence identities of the preS1-S2 regions among the different species were much lower than those of other regions (Table 1), as reported in previous studies [6,32]. Additional detailed analyses enabled us to identify unique genetic changes within each species of orthohepadnavirus. 

In this report, we discovered unique arrangements of SVs that were composed of multiple SVs at certain positions of the genome as shown in Figure 2A–G, and which we provisionally defined as SV polymorphisms. These sites could be considered as hotspots for polymorphic genetic diversity of orthohepadnaviruses. Our findings imply that the viral genomes diversified at certain positions by introduction of SV polymorphisms. The mechanism is still not clear, but two possibilities are proposed. One possible mechanism is that multiple SVs were derived from the introduction of SVs into the original prototype that did not contain any SVs. The other possibility is that combinations of deletions and insertions of SVs, which represent complex SVs, occurred in the viral genome and were maintained thereafter. 

Combinations of repeated pair-wise and multiple alignment analysis revealed highly complicated covert SV polymorphisms in the pre-S1 region. Previous reports have suggested that the complexity of genetic diversity of HBVs is related to their mosaic genome structure [15,16,17], and Littlejohn et al. [4] described the HBV genomes as a blend of small segments from the genomes of different strains rather than simply an accumulation of nucleotide substitutions. This report confirmed these previous speculations.

Lauber et al. [33] proposed that pre-S1-S2 insertions induced drastic changes to nackednavirus by providing further evolution to hepadnavirus and liver tropism. Their study implied that the pre-S1-S2 region is different from other parts of the hepadnavirus genome in its origin. Low sequence similarity of the pre-S region has been previously described by evolutionary analysis [6,32]. In addition, the pre-S1 protein is related to species specificity [34] (for example, human HBV uses pre-S1 to bind to NTCP [35]). Along with our data demonstrating that high complexity in the pre-S1 region of orthohepadnaviruses may be induced by SV polymorphisms, it is suggested that the viruses evolved, gained liver tropism by introducing pre-S insertions, and further developed to infect a variety of hosts by incorporating highly complicated genetic variations.

Regarding the correlation of SVs in HBV genome with virological and clinical characteristics, previous studies reported that complex forms of SVs in human HBV changed the transcription efficiency of pregenomic and preS/S RNA levels, and excessive expression of HBcAg in nucleus and perinucleus was observed. In addition, the SVs in HBV were observed in patients with HCC, fulminant hepatitis, and severe liver disease clinically [21,22,23]. As SVs modify activity of the virus in cells, polymorphic SVs observed in this study may affect adaptation of the virus in various hosts.

Although our analyses of SV polymorphisms in the pre-S1 region may have some limitations, the important findings that we have discovered in this study is an alternative and more plausible explanation for the genetic diversity of orthohepadnaviruses; rather than simple accumulations of nucleotide substitutions and recombinations, we propose that SV polymorphisms contributed to the viral genome diversity. Conventional concepts and methodologies have elucidated the genetic diversity of primate HBVs to a certain extent, but we believe that, due to the high complexity of the genetic diversity in other mammalian HBVs, our findings on complicated SVs in HBVs and the methods of analyses that we present here would be useful to further elucidate genetic changes in orthohepadnaviruses. Our data and further research on SVs in HBVs may help clarify the pathogenesis of these viruses in addition to understanding the genetic diversity and evolution of hepadnaviruses.

## 5. Conclusion

We have discovered unique polymorphic SVs, which we referred to as SV polymorphisms, by the analysis of multiple mammalian HBVs. Further, using a combination of pair-wise and multiple alignment analysis, we found very complicated SVs in the pre-S1 region, which were considered to be composed of SV polymorphisms.

## Figures and Tables

**Table 1:** The number of sequence gaps and sequence identities (%) in the full genome and each ORF among wooly monkey Hepatitis B virus (WMHBV), bat Hepatitis B viruses (HBVs), and rodent HBVs.

	Full	Small S	X	Core	Pre-S1-S2
Gaps	131	23	20	14	74
Identity	67.1 ± 7.1	79.5 ± 6.0	69.1 ± 7.6	69.3 ± 7.5	52.5 ± 10.0

The total number of sequence gaps in bat and rodent HBVs using WMHBV as the reference sequence was calculated from Figure 1. The number of gaps is shown. The identities (%) among WMHBV, bat HBVs (underlined strains in Table S1), and rodent HBVs (underlined strains in Table S1) were calculated using MAFFT [25], and average ± S.D. are shown.

## Supplementary Materials

The following are available online at https://www.mdpi.com/1999-4915/11/9/871/s1, Figure S1: Phylogenetic analysis of orthohepadnaviruses performed by the neighbor-joining method in 33 strains with complete genetic sequences available. Bat and rodent HBV strains used in the phylogenetic analysis are underlined in Table S1. Figure S2: Pair-wise alignments and comparisons of the WMHBV, six bat HBV and three rodent HBV sequences were performed, and 45 patterns of pair-wise data were obtained, of which the first 16 patterns are shown in Figure 3: The remaining 29 patterns are shown in Figure S2A,B. Consensus sequences were used in the analysis. This analysis was performed to clarify partial sequence identity between two species, which may indicate covert conserved genetic segments. Maintenance of % sequence identity was defined as having approximately 65% identity without gaps and a genetic sequence length ≥ 10 bps without sequence gaps, and segments satisfying these conditions are shown in vertical rectangles. Numbers above or below rectangles show the nucleotide positions where high % identity was observed in pair-wise alignment, and % identity of segments. Figure S3. Based on the pair-wise comparisons of all the strains of orthohepadnaviruses, it was found that PBHBV and BHBV-C, HBHBV and RBHBV, and three rodent HBVs showed high % identities. The nucleotide sequences were separated into segments with high and low sequence similarities with and without gaps. The segments are separated by rectangles; rectangles with solid line show the sequences with no gaps, and shaded rectangles show the sequences with gaps. Numbers below rectangles show the nucleotide positions in pair-wise analysis and % identity of segments. Detailed nucleotide sequence data of S3A, PBHBV and BHBV-C; S3B, HBHBV and RBHBV; and S3C, three rodent HBVs are shown. Table S1: Table S1. List of orthohepadnavirus sequences.
