# Supplementary material for: Novel Genetic Rearrangements Termed “Structural Variation Polymorphisms“ Contribute to the Genetic Diversity of Orthohepadnaviruses
Source: Viruses. 2019 Sep 17;11(9):871. doi: 10.3390/v11090871 (PMC6783994; doi:10.3390/v11090871)
Supplement: Supplementary file 1 [file viruses-11-00871-s001.pdf]

Supplementary Materials

Supplementary table

Table S1. List of orthohepadnavirus sequences

Species	Subclass	Number	Accession number
Human HBV			
	Genotype A	150	FM199977, GU563548, FM199979, HM535205, DQ020002, AF297621, AF297622, AY233290, AY233284, AY233281, AY233288, AY233283, AY233285, FJ692585, HE974363, AB241114, AB116091, AB453986, AB453987, AB241115, AB116088, AB076679, AB116093, AY903452, AB453989, AB453988, AY934771, AY373429, KJ638661, AY934768, AY934767, AY934769, AY934770, AY934772, AY934766, AB116089, AY233274, AF297625, AB116082, AB116087, M57663, AB076678, AB116094, AB116084, AB116086, DQ020003, EF103278, AY161141, AY373432, AF297623, AY233275, AY233276, AY233277, AY233278, AY233279, AY233282, AY233287, AY233289, AF418674, AY161140, AY161138, AB116083, U87742, EU366129, AB116085, FM199974, AY934773, AM494718, AB246335<br>AB205118, AY233280, AF297624, S50225, V00866, Z35717, X70185, EU594392, EU594390, EU594391, EU594395, EU594394, EU594384, EU594386, EU594383, EU594393, EU594388, EU594389, EU594387, EU594385, AY152726, AY902775, AB116077, AJ012207, AB116078, Z72478, AB116080, L13994, AB116079, X02763, GU563546, AB116081, AB014370, AJ344115, AF090838, X51970, AF090841, AB116076, EU859911, EU859929, GU563554, EU859942, X70185, AB064314, AF090839, AF090840,

			AF536524, AF537371, AF537372, AJ309369, AJ309370, AJ309371, AY034878, AY128092, GQ477461, EU859948 AB194952, AB194951, AB194950, AM184126, AM180623, AM184125, AY934764, FJ692613, FJ692611, FJ692554, FJ692556, FN545831, GQ331047, GQ331046, GQ331048, FN545830, FN545840, FN545839, FN545829, FN545832, FN545837, FN545828, FN545835, FN545833, FN545834
	Genotype B	40	D50521, AB010292, AB073849, AB073852, D23679, D23677, AB073846, D00329, AB073851, AB602818, AB073825, AB073831, AF121244, AB073828, AB073821, AB073833, AB073836, AF100309, AB073822, AB073834, M54923, D00331, AB031266, AB073835, AY033072, DQ463801, DQ463797, AB287314, DQ463799, DQ463787, DQ463795
	Genotype C	168	AB074756, AF068756, AB112408, AB112348, AB112066, AB112471, AB074047, AB205125, AB112065, AB112472, AB074755, AF223959, AF223956, AF223955, AY217371, AJ748098, AB111946, AB074756, AB112063, AF223954, AY167099, AY217374, AF286594, AP011097, AF223960, AF223957, JQ801478, HM011488, GQ924642, GQ377536, JQ801522, JN827423, KM999990, JQ801486, JN827416, JQ801508, HM011491, GQ924658, JQ801500, GQ924609, JQ429078, GQ924616, JQ801493, KC315399, GQ924612, JQ801470, JQ801517, GQ358153, GQ358154, AB205124, AF330110, AY641558, AY641561, AY641563, AY641562, AB205123, AB113876, AB113875, AB113877, AB115417, AY800390, AB033550, X04615, AB033553, D23684, M38636, GQ377535, EU916238, EU939592, GQ377585, AB033556, AY206378, GQ227694, EU939553, EU939536, GQ377514,

			GQ924633, EU916204, FJ899796, FJ562292, FJ562251, FJ787445, EU939557, KM999991, HM750134, EU589345, EU939647, EU589340, JQ040158, EU939587, FJ386586, X01587, AF533983, AP011098, AB202071, AB113879, AY247031, AB014362, AY057947, EU939630, GQ377539, GQ377556, GQ377604, GQ377631, GQ377549, GQ377613, GQ377590, X75656, X75665, AF241410 AB048704, AB048705, AB241109, AB241113, AP011100, AB241110, EU410081, AP011101, EU410080, JN827414, AB241112, KM999992, AB241111, JN827415, EU410079, AP011099, AB493843, AB493840, AB493847, AB493837, AB493842, AB493844, AP011102, AB493838, GQ358156, GQ358155, AB493839, AB493841, AP011103, AB554022, AB554015, AB554021, KM999993, AB554014, EU670263, GU721029, AP011105, AP011104, AP011107, AP011106, EU306671, EU306672, AP011108, AB540583, AB554019, AB554020, AB554025, AB554018, AB560661, GQ358157, AB644285, AB644281, AB644282, AB644280, AB644283, AB644284, HM011493, AB644286, AB644287
	Genotype D	79	AB104709, AB104711, AB104712, AB126581, AB188244, AB222710, AB222711, AB222712, AB222713, AB246347, AB246348, AF121240, AF121241, AF280817, AY161157, AY721605, AY721606, AY721607, AY721608, AY721609, AY721612, AY741797, AY945307, X02496, Y07587, EU594396, EU594397, AB078032, AB078033, AB090270, AB109475, AB109476, AB110075, AB116266, AB120308, AB205126, AB205127, AB210822, AB267090, AJ627220, AJ627223, AY090453, Z35716, EU594403, EU594402, EU594401, EU594400, EU594410,

			EU594409, EU594408, EU594407, EU594416, EU594415, EU594428, EU594399, EU594432, EU594431, EU594425, EU594423, EU594422, EU594421, EU594405, AJ131956, AJ344117, AY233291, AY233292, AY233293, AY233294, AY233295, AY233296, DQ111987, V01460, EU594382, EU594436, EU594434, AB033559, AB048702, AB048703, DQ315779
	Genotype E	38	AB091255, AB091256, AB201288, AB201290, AB201289, AB201287, X75664, X75657, DQ060822, FN594761, FN594764, AM494711, AM494712, AM494714, AM494706, AM494697, FN594754, GQ161783, GQ161811, AB205188, FN594749, DQ060823, DQ060825, AY935700, DQ060824, DQ060829, DQ060830, DQ060826, GQ161790, AB205192, AB194948, AM494713, AB106564, AB194947, FN594752, GQ161828, GQ161791, FN594759
	Genotype F	38	AY090459, AY090461, AY090458, AY090456, AB116552, AF223964, AY179735, AF223963, HM585199, AB116654, AB086397, EU670262, KJ638656, KJ638663, X69798, AY090455, AB116551, AB036920, AB116550, AY179734, AF223962, AB166850, AF223965, AB036915, DQ823090, DQ899148, AY311370, AB036914, AB036919, AB036916, AB036912, AB036911, AB036913, DQ899149, FJ589067, AB036910, X75663, AB116549
	Genotype G	13	AB056513, AF405706, AF160501, AB064310, AP007264, DQ207798, EF634481, AB064313, AB064311, AB056514, AB064312, AB056515, AB375170
	Genotype H	30	AY090454, AY090457, AY090460, AB059661, HM066946, AB059660, AB375161, AB375163, HM117850, AB516394, HM117851, AB064315, AB516395, AB375159, AB375160, AB516393,

			AB375162, AB059659, FJ356716, FJ356715, AB375164, AB266536, EU498228, AB353764, AP007261, AB205010, AB298362, EF157291, AB179747, AB275308
Non-Human Primate HBVs			
	Orangutan HBV	8	AF193864, AF193863, EU155825, EU155824, EU155821, EU155822, EU155827, NC_002168
	Chimpanzee HBV	27	AF242586, AF242585, D00220, AF305327, AF222323, AF222322, FJ798099, FJ798098, AB046525, AJ131575, JQ664509, JQ664508, JQ664507, JQ664506, JQ664505, JQ664504, AM117396, AM117395, AY330911, AF242585, AB032433, AB032432, AM117397, AY330912, HQ018764, AF498266, HQ018763
	Gorilla HBV	6	JQ664503, JQ664502, FJ798097, FJ798095, FJ798096, AJ131567
	Gibbon HBV	27	U46935, AB823656, AB823657, AB823658, AB823659, AB823660, AB823661, AB823662, EU155829, EU155828, AJ131568, AY781183, AY781180, AB037928, AJ131572, AJ131571, AJ131569, AY330913, AY330915, AJ131573, AY077735, AJ131574, AY330914, AJ131568, AY077736, AY330917, AY330916
	WMHBV	1	AF046996
Bat HBVs			
	TBHBV	4	KC79037, KC790379, KC790380, KC790381
	PBHBV	3	KF939648, KF939649, KF939650
	BHBV-C	3	KY905326, KY905327, KY905329
	LBHBV	3	JX941466, JX941467, JX941468
	HBHBV	1	KC790377
	RBHBV	4	KC790373, KC790374, KC790375, KC790376
Rodent HBVs			
	WHV	14	AY334075, AY628095, AY628096, GU734791, J02442, J04514, KF874491, KF874492, KF874493,

		M11082, M18752, M19183, M90520, M18752
GSHV	1	K02715
ASHV	1	U29144

Double underlined sequences in human HBV/A and HBV/C correspond to HBV/A (Del) and HBV/C (Del) in Figure 2C, respectively. Underlined bat and rodent HBV sequences are those that were used in the analysis of sequence identity shown in Table 1.

Supplementary figures

Figure S1.

Phylogenetic analysis of orthohepadnaviruses performed by the neighbor-joining method in 33 strains with complete genetic sequences available. Bat and rodent HBV strains used in the phylogenetic analysis are underlined in Table S1.

Figure S1

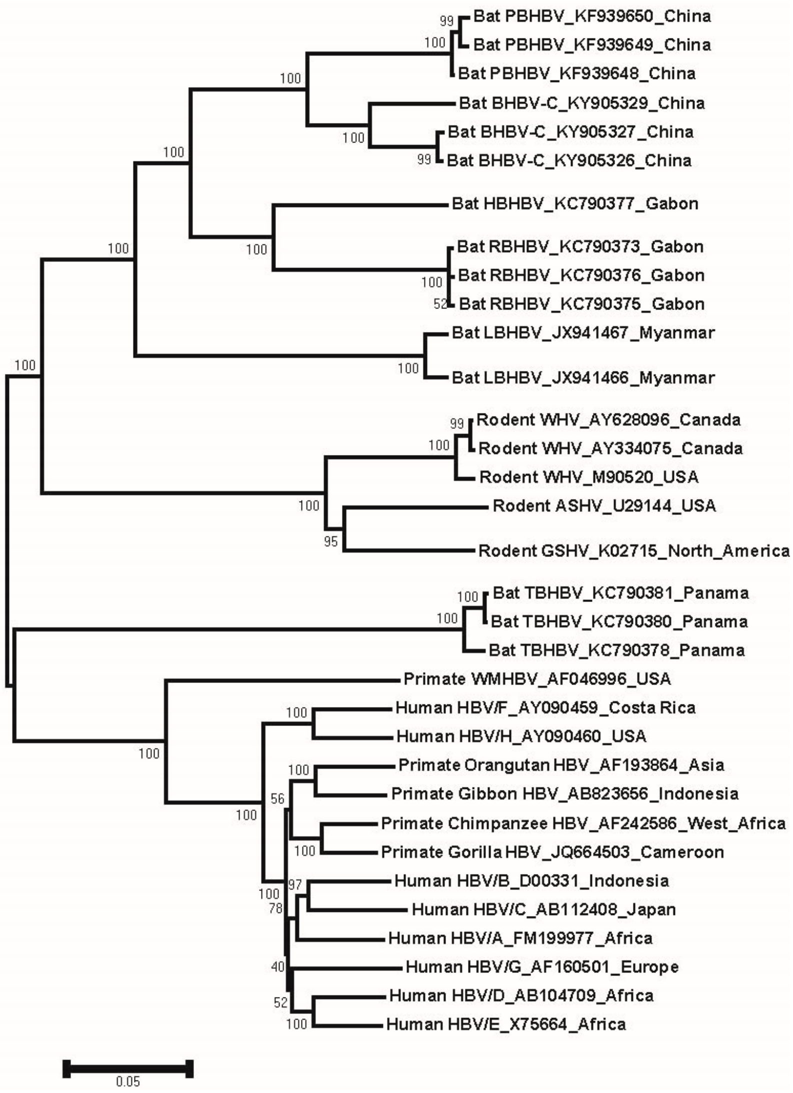

Figure S2. Pair-wise alignments and comparisons of the WMHBV, six bat HBV and three rodent HBV sequences were performed, and 45 patterns of pair-wise data were obtained, of which the first 16 patterns are shown in Figure 3. The remaining 29 patterns are shown in Figure S2A and B. Consensus sequences were used in the analysis. This analysis was performed to clarify partial sequence identity between two species, which may indicate covert conserved genetic segments. Maintenance of % sequence identity was defined as having approximately 65% identity without gaps and a genetic sequence length ≥ 10 bps without sequence gaps, and segments satisfying these conditions are shown in vertical rectangles. Numbers above or below rectangles show the nucleotide positions where high % identity was observed in pair-wise alignment, and % identity of segments.

Figure S2A

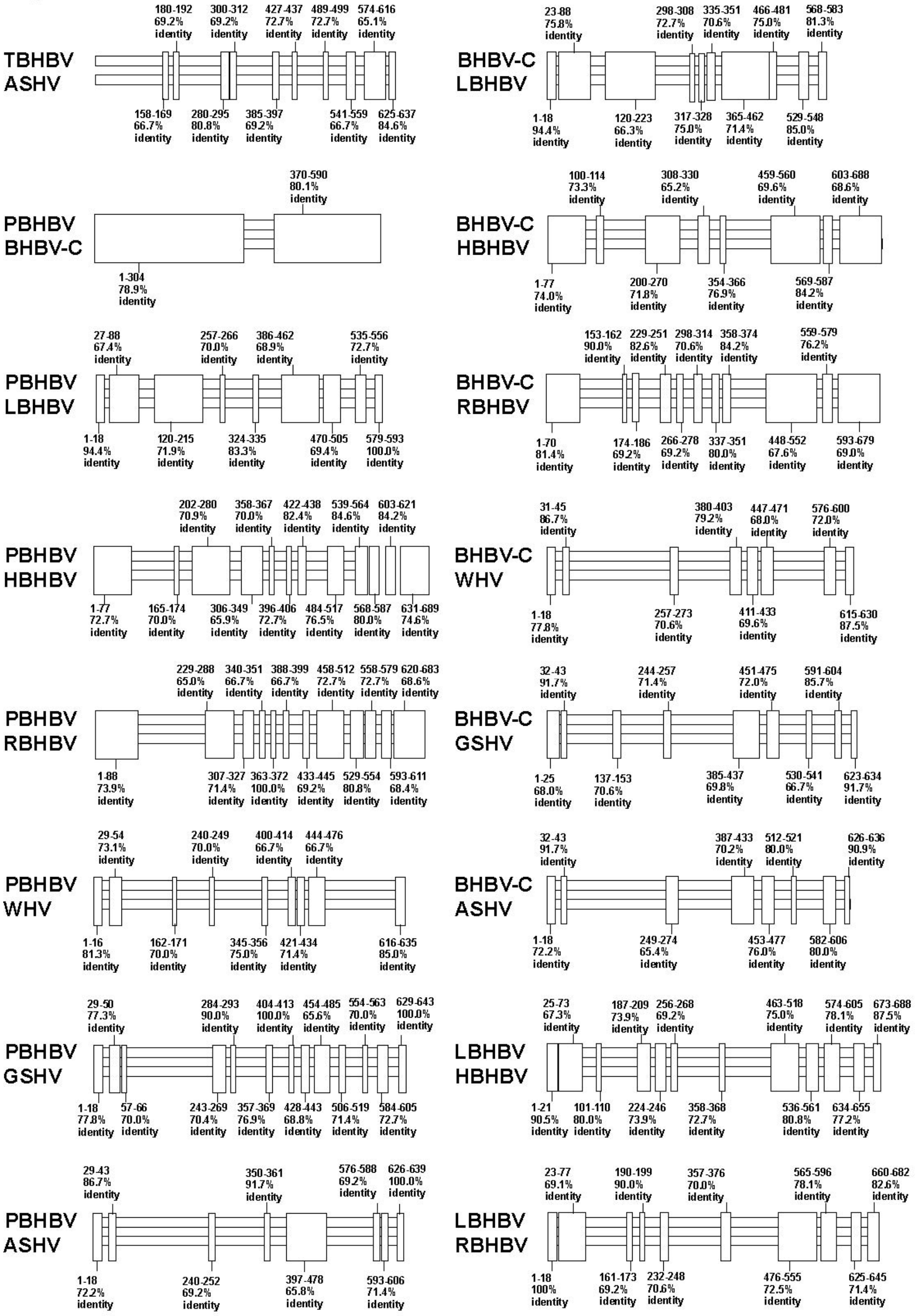
TBHBV
ASHV
180-192 69.2% identity
300-312 69.2% identity
427-437 72.7% identity
489-499 72.7% identity
574-616 65.1% identity
158-169 66.7% identity
280-295 80.8% identity
385-397 69.2% identity
541-559 66.7% identity
625-637 84.6% identity
BHBV-C
LBHBV
23-88 75.8% identity
298-308 72.7% identity
335-351 70.6% identity
466-481 75.0% identity
568-583 81.3% identity
1-18 94.4% identity
120-223 66.3% identity
317-328 75.0% identity
365-462 71.4% identity
529-548 85.0% identity
PBHBV
BHBV-C
370-590 80.1% identity
1-304 78.9% identity
BHBV-C
HBHBV
100-114 73.3% identity
308-330 65.2% identity
459-560 69.6% identity
603-688 68.6% identity
1-77 74.0% identity
200-270 71.8% identity
354-366 76.9% identity
569-587 84.2% identity
PBHBV
LBHBV
27-88 67.4% identity
257-266 70.0% identity
386-462 68.9% identity
535-556 72.7% identity
1-18 94.4% identity
120-215 71.9% identity
324-335 83.3% identity
470-505 69.4% identity
579-593 100.0% identity
BHBV-C
RBHBV
153-162 90.0% identity
229-251 82.6% identity
298-314 70.6% identity
358-374 84.2% identity
559-579 76.2% identity
1-70 81.4% identity
174-186 69.2% identity
266-278 69.2% identity
337-351 80.0% identity
448-552 67.6% identity
593-679 69.0% identity
PBHBV
HBHBV
202-280 70.9% identity
358-367 70.0% identity
422-438 82.4% identity
539-564 84.6% identity
603-621 84.2% identity
1-77 72.7% identity
165-174 70.0% identity
306-349 65.9% identity
396-406 72.7% identity
484-517 76.5% identity
568-587 80.0% identity
631-689 74.6% identity
BHBV-C
WHV
31-45 86.7% identity
380-403 79.2% identity
447-471 68.0% identity
576-600 72.0% identity
1-18 77.8% identity
257-273 70.6% identity
411-433 69.6% identity
615-630 87.5% identity
PBHBV
RBHBV
229-288 65.0% identity
340-351 66.7% identity
388-399 66.7% identity
458-512 72.7% identity
558-579 72.7% identity
620-683 68.6% identity
1-88 73.9% identity
307-327 71.4% identity
363-372 100.0% identity
433-445 69.2% identity
529-554 80.8% identity
593-611 68.4% identity
BHBV-C
GSHV
32-43 91.7% identity
244-257 71.4% identity
451-475 72.0% identity
591-604 85.7% identity
1-25 68.0% identity
137-153 70.6% identity
385-437 69.8% identity
530-541 66.7% identity
623-634 91.7% identity
PBHBV
WHV
29-54 73.1% identity
240-249 70.0% identity
400-414 66.7% identity
444-476 66.7% identity
1-16 81.3% identity
162-171 70.0% identity
345-356 75.0% identity
421-434 71.4% identity
616-635 85.0% identity
BHBV-C
ASHV
32-43 91.7% identity
387-433 70.2% identity
512-521 80.0% identity
626-636 90.9% identity
1-18 72.2% identity
249-274 65.4% identity
453-477 76.0% identity
582-606 80.0% identity
PBHBV
GSHV
29-50 77.3% identity
284-293 90.0% identity
404-413 100.0% identity
454-485 65.6% identity
554-563 70.0% identity
629-643 100.0% identity
1-18 77.8% identity
57-66 70.0% identity
243-269 70.4% identity
357-369 76.9% identity
428-443 68.8% identity
506-519 71.4% identity
584-605 72.7% identity
LBHBV
HBHBV
25-73 67.3% identity
187-209 73.9% identity
256-268 69.2% identity
463-518 75.0% identity
574-605 78.1% identity
673-688 87.5% identity
1-21 90.5% identity
101-110 80.0% identity
224-246 73.9% identity
358-368 72.7% identity
536-561 80.8% identity
634-655 77.2% identity
PBHBV
ASHV
29-43 86.7% identity
350-361 91.7% identity
576-588 69.2% identity
626-639 100.0% identity
1-18 72.2% identity
240-252 69.2% identity
397-478 65.8% identity
593-606 71.4% identity
LBHBV
RBHBV
23-77 69.1% identity
190-199 90.0% identity
357-376 70.0% identity
565-596 78.1% identity
660-682 82.6% identity
1-18 100% identity
161-173 69.2% identity
232-248 70.6% identity
476-555 72.5% identity
625-645 71.4% identity

Figure S2B

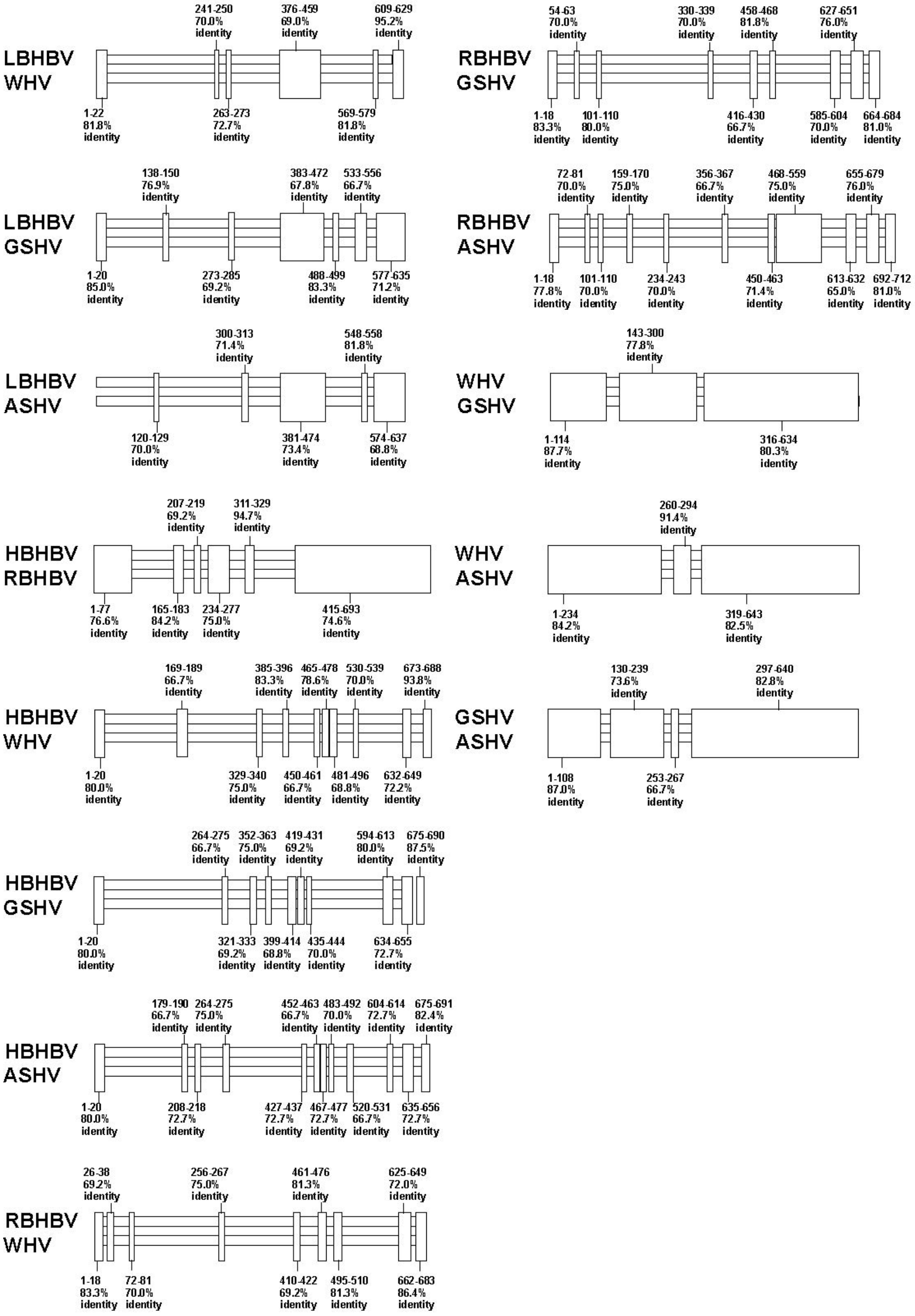

LBHBV WHV
241-250 70.0% identity
376-459 69.0% identity
609-629 95.2% identity
1-22 81.8% identity
263-273 72.7% identity
569-579 81.8% identity
RBHBV GSHV
54-63 70.0% identity
330-339 70.0% identity
458-468 81.8% identity
627-651 76.0% identity
1-18 83.3% identity
101-110 80.0% identity
416-430 66.7% identity
585-604 70.0% identity
664-684 81.0% identity
LBHBV GSHV
138-150 76.9% identity
383-472 67.8% identity
533-556 66.7% identity
1-20 85.0% identity
273-285 69.2% identity
488-499 83.3% identity
577-635 71.2% identity
RBHBV ASHV
72-81 70.0% identity
159-170 75.0% identity
356-367 66.7% identity
468-559 75.0% identity
655-679 76.0% identity
1-18 77.8% identity
101-110 70.0% identity
234-243 70.0% identity
450-463 71.4% identity
613-632 65.0% identity
692-712 81.0% identity
LBHBV ASHV
300-313 71.4% identity
548-558 81.8% identity
120-129 70.0% identity
381-474 73.4% identity
574-637 68.8% identity
WHV GSHV
143-300 77.8% identity
1-114 87.7% identity
316-634 80.3% identity
HBHBV RBHBV
207-219 69.2% identity
311-329 94.7% identity
1-77 76.6% identity
165-183 84.2% identity
234-277 75.0% identity
415-693 74.6% identity
WHV ASHV
260-294 91.4% identity
1-234 84.2% identity
319-643 82.5% identity
HBHBV WHV
169-189 66.7% identity
385-396 83.3% identity
465-478 78.6% identity
530-539 70.0% identity
673-688 93.8% identity
1-20 80.0% identity
329-340 75.0% identity
450-461 66.7% identity
481-496 68.8% identity
632-649 72.2% identity
GSHV ASHV
130-239 73.6% identity
297-640 82.8% identity
1-108 87.0% identity
253-267 66.7% identity
HBHBV GSHV
264-275 66.7% identity
352-363 75.0% identity
419-431 69.2% identity
594-613 80.0% identity
675-690 87.5% identity
1-20 80.0% identity
321-333 69.2% identity
399-414 68.8% identity
435-444 70.0% identity
634-655 72.7% identity
HBHBV ASHV
179-190 66.7% identity
264-275 75.0% identity
452-463 66.7% identity
483-492 70.0% identity
604-614 72.7% identity
675-691 82.4% identity
1-20 80.0% identity
208-218 72.7% identity
427-437 72.7% identity
467-477 72.7% identity
520-531 66.7% identity
635-656 72.7% identity
RBHBV WHV
26-38 69.2% identity
256-267 75.0% identity
461-476 81.3% identity
625-649 72.0% identity
1-18 83.3% identity
72-81 70.0% identity
410-422 69.2% identity
495-510 81.3% identity
662-683 86.4% identity

Figure S3. Based on the pair-wise comparisons of all the strains of orthohepadnaviruses, it was found that PBHBV and BHBV-C, HBHBV and RBHBV, and three rodent HBVs showed high % identities. The nucleotide sequences were separated into segments with high and low sequence similarities with and without gaps. The segments are separated by rectangles; rectangles with solid line show the sequences with no gaps, and shaded rectangles show the sequences with gaps. Numbers below rectangles show the nucleotide positions in pair-wise analysis and % identity of segments. Detailed nucleotide sequence data of S3A, PBHBV and BHBV-C; S3B, HBHBV and RBHBV; and S3C, three rodent HBVs are shown.

Figure S3A

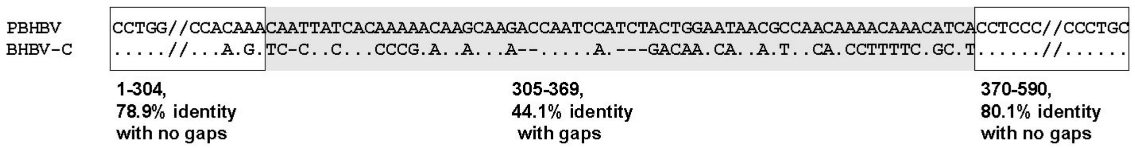

Figure S3B

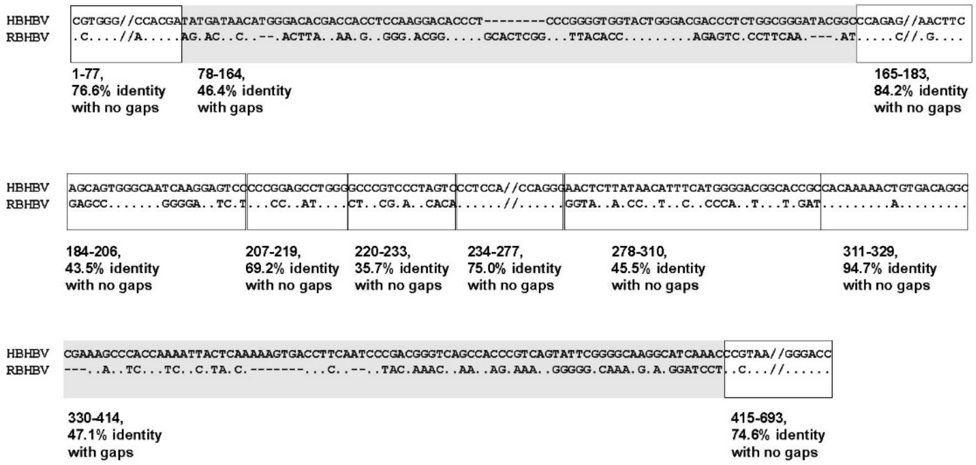

Figure S3C

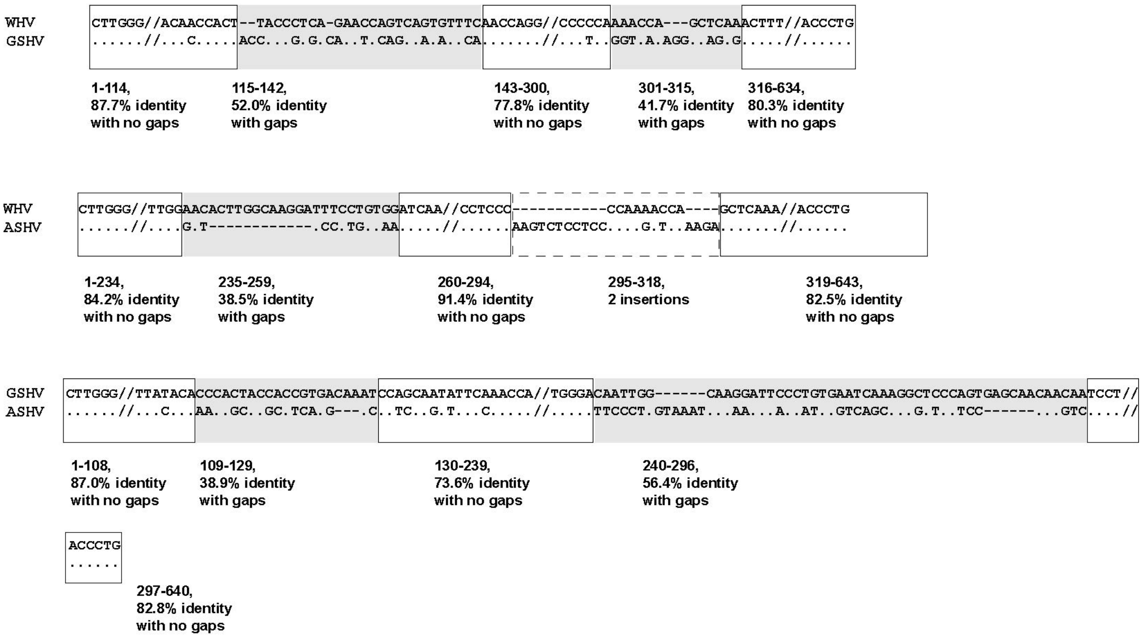
WHV
GSHV
CTTGGG//ACAACCACT--TACCCTCA-GAACCAGTCAGTGTTTCAACCAGG//CCCCCAAAACCA---GCTCAAACTTT//ACCCTG
......//...C.....ACC...G.G.CA..T.CAG..A.A..CA......//...T..GGT.A.AGG..AG.G...../......
1-114,
87.7% identity
with no gaps
115-142,
52.0% identity
with gaps
143-300,
77.8% identity
with no gaps
301-315,
41.7% identity
with gaps
316-634,
80.3% identity
with no gaps
WHV
ASHV
CTTGGG//TTGGAACACTTGGCAAGGATTTCCTGTGGATCAA//CCTCCC-----------CCAAAACCA----GCTCAAA//ACCCTG
......//....G.T------------.CC.TG..AA.....//......AAGTCTCCTCC....G.T..AAGA......//......
1-234,
84.2% identity
with no gaps
235-259,
38.5% identity
with gaps
260-294,
91.4% identity
with no gaps
295-318,
2 insertions
319-643,
82.5% identity
with no gaps
GSHV
ASHV
CTTGGG//TTATACACCCACTACCACCGTGACAAATCCAGCAATATTCAAACCA//TGGGACAATTGG------CAAGGATTCCCTGTGAATCAAAGGCTCCCAGTGAGCAACAACAATCCT//
......//...C...AA..GC..GC.TCA.G---.C..TC..G.T...C.....//.....TTCCCT.GTAAAT...AA...A..AT..GTCAGC...G.T..TCC------...GTC....//
1-108,
87.0% identity
with no gaps
109-129,
38.9% identity
with gaps
130-239,
73.6% identity
with no gaps
240-296,
56.4% identity
with gaps
ACCCTG
......
297-640,
82.8% identity
with no gaps
